# Factors Associated with Post-Traumatic Growth during the COVID-19 Pandemic: A Systematic Review

**DOI:** 10.3390/jcm13010095

**Published:** 2023-12-23

**Authors:** Andrea Bovero, Sarah Balzani, Gabriela Tormen, Francesca Malandrone, Sara Carletto

**Affiliations:** 1 Clinical Psychology Unit, University Hospital “Città della Salute e della Scienza di Torino”, 10123 Torino, Italy; abovero@cittadellasalute.to.it (A.B.); sarah.balzani@edu.unito.it (S.B.); gabriela.tormen@edu.unito.it (G.T.); sara.carletto@unito.it (S.C.); 2 Department of Clinical and Biological Sciences, University of Turin, 10043 Turin, Italy

**Keywords:** COVID-19, post-traumatic growth, trauma, protective factors, systematic literature review

## Abstract

The COVID-19 pandemic was an unprecedented event that further stimulated the debate on the concept of trauma. To increase knowledge about the traumatic potential of the pandemic, the main objective of this study was to identify, through a systematic literature review, the main factors associated with the adaptive outcome of post-traumatic growth caused by COVID-19. Studies were selected from the PsychInfo, Embase, and PubMed databases, and 29 articles were included at the end of the screening process. The identified factors are of different natures, including personal variables such as personality traits, coping, and cognitive strategies used to face adversity, and interpersonal variables, one of the most important of which is the level of social support. In addition, several results confirmed a relationship between post-traumatic growth and post-traumatic stress symptoms, as well as indices related to psychological well-being. Finally, the results are discussed by comparing them with those already present in the literature, as well as with some of the main explanatory models of post-traumatic growth. In this regard, some of the factors identified, such as maladaptive coping, avoidance symptoms, optimism, and low-stress tolerance, suggest the possibility that the process of post-traumatic growth may also be characterized by an illusory dimension.

## 1. Introduction

The COVID-19 pandemic was an unprecedented event that further broadened the trauma debate. Due to the simultaneity and multiplicity of stressors, its effects may represent a unique type of trauma that has not yet been represented within the main paradigms [1]. The main sources of stress were economic uncertainty, fear of contagion, grief, and lockdown, which resulted in the greatest psychological distress [1]. The lockdown represented a strong psychological stress factor that increased social isolation, loneliness, anxiety, boredom, insomnia, and depression [2]. Many studies have focused on the negative effects of trauma caused by the pandemic [2,3,4,5,6,7,8,9,10,11,12,13]. These effects concern both the direct impact on people’s mental health and the indirect impact that changes in lifestyle, including increased sedentariness and reduced sleep quality [8,9], have had on well-being.

Other studies have examined the presence of positive changes that occurred in people’s lives as they attempted to cope with this adverse event [14,15,16]; these positive changes can be identified with the process of post-traumatic growth (PTG). According to Tedeschi and Calhoun’s model [17], post-traumatic growth is an individual’s experience of significant positive change arising from the struggle with a major life crisis. These changes result from the attempt to cope with a seismic event that has resulted in the shattering of one’s cognitive schemas [18]. People who go through this type of traumatic experience develop beyond their previous level of adaptation or psychological functioning and therefore experience growth [17]. These authors explore personality traits related to growth, emphasizing creativity in problem-solving and the ability to regulate behavior in the external environment [19]. Following a challenging event, individuals undergo emotional, cognitive, and behavioral shifts, enteing a phase of rumination and adopting emotion-based coping strategies, leading to eventual growth marked by improved discomfort management, serenity, self-awareness, increased life appreciation, and enhanced creativity in behavior [19]. In the ‘Janus Face of Self-Perceived Growth’ model [20], also based on the work of Taylor et al. [21], it is hypothesized that, in addition to this dimension of real growth, there may also be illusory growth, which can be both functional and dysfunctional in relieving the stress caused by trauma [20].

For the purposes of this study, it is important to clarify the concept of trauma in the pandemic context, and in particular its meaning within the notion of PTG, as opposed to the meaning it assumes in relation to other trauma outcomes, such as PTSD. The latter is based on a conceptualization of trauma that requires “actual or threatened death, serious injury, or sexual violence” [22]. In contrast, Tedeschi et al. [23] merge the construct of PTG with a notion of trauma that focuses on the individual’s subjective reaction rather than the characteristics of the event itself. Indeed, according to the authors, the traumaticity of an event would be a function of the destructuring effect it has on the individual’s cognitive schemas. Therefore, when comparing these types of trauma-related outcomes, it is important to consider that they are based on trauma constructs with significant differences.

Several studies have investigated the factors associated with PTG caused by different types of traumas, such as in breast cancer patients, college students abused in childhood, and male rectal cancer patients [24,25,26]. The analysis of sociodemographic factors can also help to understand how people in more vulnerable groups, ethnic minorities, and people with mental disorders or other chronic illnesses, respond to trauma in terms of adaptive mechanisms, such as post-traumatic growth [27]. Prior research has highlighted that individuals from minority backgrounds tend to express higher levels of post-traumatic growth than their counterparts in majority groups [28,29]. Earlier studies have demonstrated that minority groups often report more adverse effects in the context of COVID-19 when compared to majority groups [30,31]. Vulnerability can also play a role in fostering positive outcomes. To put it differently, navigating the challenges of the current crisis from a standpoint of vulnerability might present an opportunity to uncover personal and environmental resources, ultimately promoting growth [23].

Regarding COVID-19 trauma, a systematic review of the literature was conducted to identify the factors associated with PTG in healthcare workers [32]. This population was one of the most vulnerable during the pandemic due to their exposure to direct and indirect trauma.

To increase knowledge of the traumatic effects of COVID-19 and to examine the protective factors associated with the adaptive outcome of post-traumatic growth, this systematic literature review aimed to synthesize studies investigating the factors associated with COVID-19-induced PTG in the general population; the only subpopulation that was excluded was that of healthcare providers, as they have already been studied [32]. Furthermore, given the duration of the pandemic, individuals may also have experienced stressors unrelated to the pandemic context, such as illness or the loss of a loved one. For this reason, only studies that used a measure of PTG that was able to capture the positive changes that individuals reported as directly related to the pandemic context were included in this review.

This review addresses a significant gap in the scientific literature on the impact of the COVID-19 pandemic on mental health. While other systematic reviews and metanalyses [13,33,34] have focused on summarizing the factors associated with adverse COVID-19 outcomes, such as PTSD, this review broadens the perspective by compiling the factors associated with the adaptive outcome of pandemic-induced PTG. Furthermore, investigating these factors in the wider population allows for the identification of a plethora of data that greatly aid the comprehension of the PTG process, as well as the wider effects of trauma. Understanding these data is crucial due to the complexity and diversity of factors associated with this positive outcome, which makes developing protocols for the active promotion of PTG challenging. Thus, it is imperative to undertake research to comprehend these factors and facilitate the implementation of practices that mitigate the adverse effects of trauma and encourage positive changes.

## 2. Materials and Methods

This systematic review was carried out following the PRISMA Statement. The protocol was registered on the PROSPERO database (Record ID: CRD42023398006).

### 2.1. Search Strategy

Searches were conducted in the following databases on 23 December 2022: PubMed, PsychInfo, and Embase. The keywords and text words used in the search for each of the considered databases were “posttraumatic growth”, “positive change”, “PTG” and “stress-related growth”. See Supplementary Material S1 for details of the search strategy. Reference lists of included articles and previous reviews were also searched.

### 2.2. Eligibility Criteria

All studies that investigated any factor associated with PTG caused by COVID-19 in the general adult population (18+) were included. The PTG scale had to be validated, standardized, and adapted to the context of COVID-19. Scale adaptation required that questionnaire instructions or items were modified to capture the positive changes that individuals reported as a result of the specific context of COVID-19. Studies had to have been published in English or Italian during the pandemic period and have a cross-sectional, longitudinal, or RCT design. Studies not meeting these criteria were excluded. Studies conducted on healthcare workers were also excluded.

### 2.3. Study Selection

Two authors (S.B. and G.T.) independently conducted standardized assessments to determine study eligibility according to the inclusion criteria. The abstracts were first screened, and all the full texts of all potential contributions were analyzed. Any disagreement was discussed with the other two authors (S.C. and A.B.) before agreement was reached. See Supplementary Material S2 for the study selection flowchart.

### 2.4. Data Extraction

Data from the selected studies were inserted into a standard template. Extracted data covered the year of publication, study design, population type, nationality, sample size, data collection period, mean age, sex, PTG assessment measure, instruments used to measure the associated factors, and the main outcomes related to PTG. Where available, information on COVID-19 prevention and control measures in place at the time of data collection was also included.

### 2.5. Quality Assessment

To assess the methodological quality and the internal validity in terms of the risk of bias (ROB) of the included studies, the Joanna Briggs Institute (JBI) critical appraisal checklist for analytical cross-sectional study was used [35]. The JBI checklist is composed of eight items regarding inclusion criteria, an adequate description of the subject and setting, appropriate measurement of exposure, criteria for measurement of the condition, the identification of confounding factors and strategies to deal with, the reliability of outcomes measures, and the appropriateness of the statistical analysis, with each item answered as “yes”, “no”, “unclear” or “not applicable”. For classification purposes, when the total number of “yes” ranged from 0 to 2, the study was classified as having high ROB; from 3 to 5, the study was classified as having a moderate ROB; and when this number was at least 6, the study was classified as having a low ROB. Two independent researchers of the review team assessed ROB, and differences were resolved through discussion among all the authors.

### 2.6. Study Synthesis

The results of the evidence found are presented as a narrative synthesis. After reading and thoroughly examining the main associations reported in the included studies, several key themes were identified, which supported the organization and comparison of the results.

## 3. Results

The PRISMA flowchart describing the selection process, including reasons for exclusion, is presented in Supplementary Material S2. The initial search retrieved 866 articles after duplicate removal. Of these, only 29 articles were considered eligible for inclusion in this review. Table 1 provides an overview of the main data extracted from the studies. Of the 29 articles included, 18 were cross-sectional, 9 were longitudinal, and 2 were prospective cohort studies with cross-sectional data. All the articles included were written in English. Seventeen articles used the PTGI-21 measurement scale, eight used the PTGI-SF scale, two used the PTGI scale, one used the PTGI-13 scale, and one used the PTGI-S scale. The studies used samples from different populations, including workers, students, people with psychiatric diagnoses, veterans, people infected with COVID-19, pregnant women, and people who had experienced bereavement due to COVID-19. The factors associated with PTG examined in the studies are multiple and heterogeneous and were therefore divided into five categories: sociodemographic factors, COVID-19-related factors, individual factors, relational factors, and factors related to psychological well-being or distress.

### 3.1. Association between PTG and Sociodemographic Factors

A relationship between PTG and ethnicity has been found in several studies [39,44,54,58]. In particular, Asians were less likely to report PTG [44], whereas in a sample of Arab and Jewish women living in Israel, Arab women experienced higher levels of growth [39]. Finally, Caucasian/European Americans reported lower levels of growth [58]. In contrast, another study measured higher levels of PTG in the non-white ethnic group [54]. Also, in Northfield and Johnston’s study [55], although ethnicity was not a significant predictor of PTG, a group difference was evident between African American and Caucasian participants, with African American participants reporting higher levels. Considering age and sex/gender, no significant differences in relation to PTG were reported in some studies investigating these variables [37,40,46,49,50,59,62,63]. In contrast, in the study by Celdrán et al. [38] and Fino et al. [42], women reported higher levels of growth, but of all the sociodemographic variables considered in the study by Celdrán et al. [38], only younger age significantly correlated with higher PTG, and the same was found in the study by Northfield and Johnston [55]; by contrast, in the study by Na et al. [54], being female was associated with pandemic growth. In the study by Ikizer et al. [46], only the level of education was a predictor of PTG, as higher levels of education were associated with lower levels of PTG, while being younger was only associated with negative outputs, such as higher levels of pandemic-related stress. In contrast, in the study by Lau et al. [50], having a university degree was associated with higher odds of PTG, as was having a higher monthly household income than the sample mean. In the longitudinal study by Landi et al. [48], being older was negatively correlated with PTG. In the study by Yan et al. [64], of all sociodemographic variables, only patients grouped based on their place of residence had a significant difference in PTG, i.e., those living in non-urban areas had higher levels than participants living in urban areas. Finally, in the study by Xiao et al. [61], having children was significantly associated with PTG, and in particular in the study by Chasson et al. [39], being primiparous was associated with higher PTG.

### 3.2. Association between PTG and COVID-19-Related Factors

In the study conducted by Celdrán et al. [38] and Hyun et al. [44], PTG showed a significant association with COVID-19 experience. However, only individuals who were infected with COVID-19 demonstrated significantly higher scores on the PTGI-SF. Anxieties and worries related to COVID-19 were associated with higher PTG [39,44], as was fear of contracting COVID-19 [53]. Similarly, in the study by Na et al. [54], most of the variance in pandemic-related PTG was explained by concerns about the effect of the pandemic on one’s physical and mental health. In the study by Fino et al. [42], fear of COVID-19 was associated with both positive mental health outcomes (post-traumatic growth) and negative outcomes (depression and anxiety), and these relationships were mediated by the type of coping strategy used. In the longitudinal study by Landi et al. [48], COVID-19 infection during the study period was positively associated with PTG at time 2 (9–19 October 2020) but not at Time 1 (9–19 July 2020). In the study by Lewis et al. [51], higher PTGI-SF scores were strongly associated with the perception of the pandemic as traumatic. Specifically, being with a family member or close friend who experienced quarantine or who tested positive for COVID-19 was correlated with a higher likelihood of PTG [50]. In the study by Xiao et al. [61] involving adult patients cured of COVID-19 and discharged from the hospital between 1 February and 30 April 2020, significant protective factors of PTG included the clinical classification of COVID-19 infection at hospital admission. Consistent with this result, in Yan et al.’s study [64], the time from onset to diagnosis was negatively correlated with PTG, indicating that shortening this process could help improve patients’ PTG. Finally, a study examined the relationship between prolonged grief (PG), post-traumatic stress (PTS), and PTG in individuals who have suffered bereavement due to COVID-19 [40]. The results showed that when the deceased was older, individuals were more likely to report high levels of PTS and PG. In contrast, conflicts in the relationship with the deceased person led to a lower likelihood of being in the group with high levels of growth and low levels of PTS and PG. At the same time, the loss of a younger loved one and the presence of a close relationship with the deceased increased the likelihood of experiencing moderate or high levels in all three dimensions.

### 3.3. Association between PTG and Individual Factors

Regarding the association between PTG and individual characteristics, several studies have revealed a significant association with the process of deliberate rumination [36,46,58,59]. A relationship was also found between the employment of cognitive reappraisal and cognitive flexibility with PTG [48,49,52]. Specifically, the connection between PTG and cognitive flexibility was solely detected in those who have reported high levels of post-traumatic stress [48]; cognitive reappraisal, coupled with social support, mediated the relationship between agency thinking and PTG [49]; and cognitive reappraisal, together with positive coping, mediated the relationship between psychological resilience and PTG [52]. In this regard, another study has also confirmed the relationship between resilience and growth [44]. Another important individual characteristic that interacts with PTG is the type of coping strategy used. In the study by Kalaitzaki et al. [47], during the first lockdown in Greece, PTG was associated with both adaptive coping strategies, i.e., positive reframing, religious coping, and the use of emotional support, and maladaptive coping strategies, i.e., self-blame, denial, and substance use; in contrast, during the second lockdown, this association was observed only with adaptive coping strategies. In contrast, in Fino et al.’s study [42], the use of a positive coping strategy (engagement coping) positively mediated the effect of fear of COVID-19 on PTG, whereas negative coping (disengagement coping) mediated the effect of fear of COVID-19 on anxiety and depression. Also, in the study by Yan et al. [64], the use of positive coping strategies was associated with higher PTG levels. Finally, in the study by Xie and Kim [62], a correlation was found between PTG and problem-focused coping, emotion-focused coping, and social-support coping while avoidance coping mediated the relationship between conscientiousness, agreeableness, and PTG. Regarding other personality traits, the study by Casali et al. [37] revealed that out of the six character-related virtues (wisdom and knowledge, courage, humanity justice, temperance, and transcendence), only humanity was significantly associated with PTG. Optimism and self-compassion [39], self-transcendence [63], agreeableness [54,62], self-esteem [64], and sense of coherence [50] were also associated with higher levels of PTG. Other personality traits associated with PTG were extraversion, conscientiousness, and emotional stability [62]. Protective psychosocial characteristics associated with PTG included religiosity/spirituality, purpose in life, and PTG compared to previous trauma [54]. The study by Shigemoto et al. [58] also revealed that those who identified as religious tended to experience higher levels of PTG. Wang and Huang’s study [60] showed that PTG significantly and positively influenced university students’ entrepreneurial intention and that self-efficacy and prosocial tendencies mediated this relationship. In the study by Xie et al. [63], the results showed that COVID-19-related PTG was a positive factor in university students’ life satisfaction and that personal values played a mediating role in this relationship. Among the predictors of PTG, the centrality of the event was found to be a significant factor [59]. Regarding the variables most related to the pandemic, the study by Xiao et al. [61], conducted among patients hospitalized for COVID-19, revealed an association between self-stigmatization and PTG. Finally, intolerance of uncertainty was associated with higher levels of PTG only in Jewish women (but not in Arab women) and in women with higher levels of self-compassion [39], whereas tolerance of distress was associated with lower levels of PTG [44].

### 3.4. Association between PTG and Relational Factors

With regard to interpersonal variables, in the studies by Wall et al. [59] and Xiao et al. [61], social support was found to be a significant protective factor for the development of PTG. The role of social support in the ability to cope with trauma and bring about positive change was also confirmed in a sample of people discharged from the hospital after hospitalization for COVID-19 [64]. In Kalaitzaki et al.’s study [47], perceived emotional social support during the first lockdown and perceived instrumental social support during the second lockdown were predictive of growth during both lockdowns. However, the results of Celdrán et al. [38] showed that a state of increased contact with others was not necessarily associated with PTG. In fact, both increased and decreased perceived loneliness were significantly associated with PTG. In addition, the authors found that an increase in meaningful conversations was associated with growth. In the study by Northfield and Johnston [55], only social support from family or friends was associated with PTG, whereas this relationship was not observed for other significant others. Hyun et al. [44] also found a positive association between PTG and family connectedness. In the study by Matos et al. [53], social connectedness, in terms of compassion and perceptions of social safety, predicted higher levels of PTG. The impact of the social dimension is manifested not only in the level of support received but also in how socially active one feels; indeed, an association was found between social engagement before and during the pandemic and perceptions of growth [49]. Interpersonal trust, i.e., rating others as trustworthy, was also associated with all dimensions of PTG [41]. Only one of the included studies focused on the relationship between work context and PTG [57]. The results showed that perceptions of the psychosocial safety climate had a positive and direct effect on PTG. Finally, at the macro-social level, the “social” dimension of growth (i.e., relating to others) fully mediated the national identity–interpersonal trust association [41].

### 3.5. Association between PTG and Factors Related to Psychological Well-Being and/or Distress

Regarding the relationship between PTG and mental health outcomes, the study by Hyun et al. [44] showed that PTSD symptoms were significant predictors of higher PTG levels; the study by Na et al. [54] revealed that pandemic-related PTSD avoidance symptoms explained a significant proportion of the variance in pandemic-related PTG; and the study by Yan et al. [64] revealed a positive correlation between PTG and PTSD in discharged COVID-19 patients, and patients with high PTSD exposure had higher PTG. In general, several studies have confirmed an association between post-traumatic stress symptoms (PTSS) and PTG experienced during the pandemic [36,46,47,48]; in addition, the study by Ikizer et al. [46] revealed an association between post-traumatic depreciation and PTG. The relationship between PTSS and PTG also appears to be influenced by other factors. Northfield and Johnston [55] found a strong positive relationship between psychological distress and growth that was mediated by social support, such that the relationship was stronger at higher levels of social support. Lau et al. [50] found that PTG was more likely in participants with high levels of post-traumatic stress but also a high sense of coherence. The results of the study by Chen and Tang [40] identified four profiles of prolonged grief, post-traumatic stress, and post-traumatic growth in people who had suffered bereavement due to COVID-19; two of these profiles were characterized by moderate and high levels of both PTG and PTS. The study by Pietrzak et al. [56] showed that the prevalence of PTG was higher in veterans with PTSD symptoms and that positive changes in appreciation of life and social relationships (two dimensions of PTG) related to the pandemic were associated with a significant reduction in the likelihood of suicidal ideation. In the study by Lewis et al. [51], higher scores on the PTGI were associated with higher levels of psychological well-being, and in the subsample of participants who perceived the pandemic as traumatic, PTG was associated with higher PTSD scores. Casali et al. [37] also confirmed the role of PTG in mental health, as it mediated the relationship between positive personality traits and psychological well-being. Furthermore, depressive symptoms were predictors of lower levels of PTG in the study by Hyun et al. [44], whereas in the study by Hyun et al. [45], it was observed that at low levels of pandemic-related stress, depressive symptoms were similar for young adults with low, moderate, or high levels of PTG. However, at high levels of pandemic-related stress, young adults with low PTG had the highest levels of depressive symptoms, whereas those with high PTG had the lowest levels of depressive symptoms. In addition, PTG appeared to play a protective role against depressive symptoms, as it attenuated the effect of COVID-19-related stress in 2020 on depressive symptoms in 2021. Finally, in the study by Goutaudier et al. [43], the highest levels of PTG were found in participants who reported moderate levels of anger, fear, and happiness during the first lockdown, whereas in the study by Yan et al. [64], a negative association was observed between PTG and anger.

### 3.6. Quality Assessment of the Included Studies

Notably, 3 studies (10.34%) had a low ROB, while 26 studies (89.65%) had a moderate ROB. None of the studies demonstrated a high ROB. Regarding study participants’ recruitment and sampling procedures, 12 studies (41.37%) did not report enough information. Three studies (10.34%) did not appropriately describe the study subjects and research setting. In all studies, adequate data analyses were carried out. Details about the results concerning the ROB assessment of the included studies are summarized in Table 2.

## 4. Discussion

To the best of our knowledge, this is the first systematic literature review that has synthesized studies investigating the factors associated with PTG induced by COVID-19. Among the sociodemographic variables, the synthesis of the studies showed already known associations, including those with young age [65] and ethnic minority status [28,29]. With regard to age, the fact that being younger is associated with higher levels of growth is also consistent with the model proposed by Tedeschi and Calhoun [18], according to which young people may be more open than older people to the learning and change that enables the growth process. Notably, one study found that the Asian American population was less likely to develop PTG than the American population [44]. This finding can be interpreted in the specific context of COVID-19, where people of Asian origin have experienced stigma and discrimination, which may have affected their mental health and ability to seek help [66], and consequently, their ability to experience positive change. The synthesis of studies on the association between PTG and income and education levels showed contradictory results. Ikizer et al. [46] found that PTG was linked to lower educational attainment and anticipation of financial risk, and higher education to lower levels of PTG, PTS, and PTD. By contrast, Lau et al. [50] found that PTG was linked to tertiary education and higher economic income, and on this basis, they suggest that education and higher income may serve as practical coping resources, in addition to psychological ones, and may mediate the relationship between stress and PTG. These disparities may be clarified by Tedeschi and Calhoun’s model [18]. If the process of PTG necessitates a “seismic” event that impacts individuals’ lives, leading to the questioning of significant mental patterns, it may be hypothesized that, while higher education and its work-related implications may have corresponded to reduced stress and mitigated the effect of the pandemic on mental health during COVID-19, the level of education was not always a protective factor due to the numerous stressors caused by the pandemic. In this regard, one of the factors associated with PTG is the centrality of the event, i.e., how critical a particular event is to a person’s identity and life story; the greater the centrality, the greater the level of growth [59]. Considering the interplay of these sociodemographic variables, it is crucial to highlight that numerous factors need to be considered to comprehend the varied reactions toward extremely demanding circumstances, such as the pandemic context.

Another interesting finding in the context of the pandemic is the association between PTG and increased loneliness [38]. Despite many studies confirming the importance of social support in supporting the growth process, as discussed by the authors, this finding opens the possibility that a change in the relational dimension, whether in terms of increased or decreased loneliness, could stimulate growth. Such a change could challenge one’s schemas and support the growth process.

In addition to the social dimension, many individual factors have been associated with PTG. Understanding these factors has important clinical implications for trauma interventions. This knowledge enables the advancement of strategies and procedures aimed at fostering PTG within the therapeutic alliance, a factor that research has demonstrated to be closely linked to psychological well-being. In this regard, the study by Pietrzak et al. [56] revealed that although the prevalence of PTG was higher in veterans with PTSD symptoms, positive changes in life evaluation and social relationships related to the pandemic were associated with a significant reduction in the likelihood of suicidal ideation. The association with PTSS and psychological distress is consistent with the possibility that the process of PTG requires a significant threat or the shattering of fundamental schemas, often accompanied by significant psychological distress [18]. Indeed, the association observed across multiple studies between PTG and concerns related to COVID-19 underscores the traumatic impact of the pandemic and the unsettling influence it exerted on individuals’ mental well-being. Furthermore, the context of COVID-19 has accentuated the range of reactions to trauma, including those that may initially seem contradictory to one another. Indeed, the study by Ikizer et al. [46] showed an association between PTG and post-traumatic depreciation. Existing literature data indicate that this aspect may manifest separately from PTG and within the same domains [67,68]. Such an association in the pandemic context suggests that the complexity of trauma may lead to both positive and negative changes in the same dimensions, which are related to each other. Nevertheless, more data are needed to clarify the relationships between these forms of trauma.

Furthermore, as a speculative hypothesis, we suggest that some factors emerging from the synthesis of the studies indicate the potential presence of an illusory dimension within PTG, consistent with the concept of the “Janus Face of Self-Perceived Growth” [20]. In particular, the literature underlines a positive association between PTG and maladaptive coping, avoidance symptoms, avoidant coping, and optimism traits, and a negative association with distress tolerance. Although these factors may represent initial attempts to alleviate the stress caused by a sudden event without negative consequences, the association with avoidance symptoms and coping may have negative effects on individual adjustment [20].

Finally, it is important to consider that few studies have reported clear data on the prevalence of PTG, which complicates the interpretation of the results. A comparison of the studies that have reported these data reveals that the prevalence of this phenomenon does not seem to be uniform across the samples studied.

This systematic literature review has several limitations that need to be considered when evaluating the results. Firstly, all studies used a self-report scale to measure PTG; therefore, there is a risk that participants may have under- or overestimated the information. At the same time, some studies used non-standardized measures for the associated factors, which are less sensitive in capturing the phenomenon of interest and hinder an accurate comparison of the factors related to PTG, as well as a clear understanding of the variables associated with the growth process. Therefore, there is a need to use more standardized measurement tools that can effectively capture the desired outcome. Secondly, the data collection process required participants to complete the PTG questionnaire with a focus on positive changes because of the pandemic. Although this criterion allowed for a better understanding of the specific effects of COVID-19, participants may have found it difficult to distinguish between specific changes caused by the pandemic and other positive developments in their lives not related to the pandemic. In addition, it is important to consider the limitations of the cross-sectional nature of most of the selected studies, including the difficulty of interpreting the associations identified and the inability to examine the temporal relationship between outcomes and other factors [69]. Finally, although all included studies were conducted during the pandemic, there were periods of heightened crisis due to increased contagion and restrictions, alternating with periods of lower tension. This may make it difficult to compare and interpret results, especially when considering PTG as a process and therefore sensitive to trauma exposure times. In this respect, most studies did not use time-sensitive measures of adverse experiences related to COVID-19. The limitations highlighted in this review underscore the importance of adopting an end-to-end research approach, emphasizing the need for a meticulous consideration of measurement tools, data collection methods, and the temporal dynamics of trauma exposure to ensure more standardized and robust analyses in future investigations of post-traumatic growth associated with the COVID-19 pandemic.

## 5. Conclusions

This systematic literature review provides important data for understanding the effects of the COVID-19 pandemic and, more generally, the effects of trauma and their association with PTG. This review suggests important future research directions for understanding the phenomenon of PTG. It is fundamental to investigate whether the currently available measures can detect real rather than illusory growth, and how different forms of response to trauma interact with each other. Finally, the synthesis of the studies showed a wide heterogeneity of protective factors that should be considered when implementing interventions to support people with traumatic experiences.

## Figures and Tables

**Table 1:** Overview of the main data extracted from the studies.

Authors	Study Design	Sample	Period of Data Collection	Mean Age	Gender	PTG Instrument	Associated Factors Instruments	Main Results on PTG	PTG Prevalence
Bayless (2021) [36]	cross-sectional study	US, Amazon Mechanical Turk (MTurk, N = 150) and undergraduate students (N = 16)	from 19 May 2020 (the US had 23,405 new COVID-19 cases per day) to 30 June 2020 (the US had 43,644 new cases per day)	35.8	38.6% females 61.4% males	PTGI 21-item COVID-19-adapted	Patient Health Questionnaire-2 (PHQ-2); Event-Related Rumination Inventory (ERRI); Form A of the Multidimensional Health Locus of Control scale (MHLC-A); PTSD Checklist for DSM-5 (PCL-5)	There was not a significant interaction between internal and external health-related locus of control domains in relation to PTGI scores; PTSS and rumination were associated with positive growth scores.	not reported
Casali et al. (2022) [37]	longitudinal study	Italy, general population (N = 254)	T1: April 2020 (first national lockdown) T2: December 2020 to January 2021 (second wave of the pandemic)	36.1	78.75% females 21.25% males	PTGI 21-item COVID-19-adapted	General health questionnaire-12 (GHQ-12); Values in action inventory of strengths-120 (VIA-IS-120)	Character had a significant direct effect on mental health at Time 2, and an indirect effect through the mediation of PTG (small); humanity was significantly related to PTG; no significant age or gender-related differences emerged in relation to PTG.	not reported
Celdrán et al. (2021) [38]	cross-sectional study	Spain, senior (55+) university students (N = 1009)	from 8–24 May 2020 (immediately after the forced lockdown in Barcelona)	66	61.7% females 38.3% males	PTGI-SF COVID-19-adapted	Series of questions regarding the Impact of COVID-19 (yes/no) and social resources (increase, no change, or decrease)	PTG was significantly associated with the experience of COVID-19, but only those who had been infected by it scored significantly higher on the PTGI-SF; age (being younger) was related to PTG; the presence of significant conversations and changes in loneliness (either an increase or a decrease) were related to PTG.	From moderate to high PTG in 20.5% of the sample
Chasson et al. (2022) [39]	cross-sectional study	Israel, Jewish, and Arab pregnant women (N = 916)	5 July to 7 October 2020 (second wave of the pandemic)	28.2	100% females	PTGI 21-item COVID-19-adapted	Intolerance of Uncertainty Scale-Short Form (IUS-12); The Life Orientation Test (LOT); The Self-Compassion Scale-Short Form (SCS-SF); COVID-19-related anxieties were measured by means of 2 items	Higher optimism and self-compassion were related to higher PTG; younger age and greater COVID-19-related anxieties were associated with higher PTG: higher fear of being infected and concern for the economic damage both significantly associated with higher PTG; being primiparous contributed to higher PTG; a positive association between intolerance of uncertainty and PTG was found among Jewish, but not Arab women; positive association between intolerance of uncertainty and PTG was stronger among women reporting higher self-compassion.	not reported
Chen and Tang (2021) [40]	cross-sectional study	China, people bereaved due to COVID-19 (N = 422)	September and October 2020 (because of restrictions in social contact, after a person died from COVID-19, family members were usually not able to gather together to attend the wake preceding the funeral)	32.7	44.5% females 55.5% males	PTGI 21-item COVID-19-adapted	International ICD-11 Prolonged Grief Disorder Scale (IPGDS); Post-Traumatic Stress Disorder Checklist for DSM-5 (PCL-5)	Four profiles of prolonged grief, post-traumatic stress, and PTG were identified; those who were bereaved of an older loved one were more likely to be in the growth group rather than the moderate-combined group vs. death of a younger person was more likely to cause a moderate-combined than a growth profile; a closer relationship perceived by the bereaved increased the likelihood of being in the high-combined group and conflicts in the relationship decreased the chance of ending up in the most adaptive group of growth.	from moderate to high PTG in 90% of the sample
Ellena et al. (2021) [41]	cross-sectional study	Italy, young adults (N = 2000)	between 27 and 31 March 2020 (during the peak of the COVID-19 crisis in Italy)	27.1	49% females 51% males	PTGI COVID-19-adapted	In-Group Identification Scale adapted; A single item adapted from Zmerli and Newton (2008) was used to measure levels of interpersonal trust: “Since the start of COVID-19 emergency, how has your attitude toward this statement changed? Most people are trustworthy ” (10-point Likert); Trust in institutions was measured by asking: “Since the beginning of COVID-19 emergency how has your confidence in these institutions changed ?” (5-point Likert)	National identity scores were positively related to interpersonal trust and the five PTG dimensions; each PTG dimension was positively associated with interpersonal trust; PTG “relating to others”, perceptions of having new possibilities, and spiritual change dimensions mediated the positive relationship between national identity and interpersonal trust, whereas personal strength and appreciation of life did not.	not reported
Fino et al. (2022) [42]	cross-sectional study	Albania, general population (N = 231)	from 16 to 30 December 2020 (lockdown)	39.9	73.2% females 26.8% males	PTGI 21-item COVID-19-adapted	Coping Strategies Inventory Short-Form (CSI–SF); Hospital Anxiety and Depression Scale (HADS); Single items asking respondents to indicate their level of fear on a Likert scale; Adaptation of 8 items from the SARS Fear Scale (SFS)	Fear of COVID-19 was associated with both stress and growth outcomes, and this relationship was moderated by trait resilience; engagement coping was the only significant mediator of the relationship between COVID-19 fear and PTG.	not reported
Goutaudier et al. (2022) [43]	longitudinal study	France, general population (N = 1075)	T1: March to May 2020 (first lockdown) T2: March to May 2021	47.5	62.4% females 37.6% males	PTGI 21-item COVID-19-adapted	Beck Depression Inventory Short Form; Spielberger State–Trait Anxiety Inventory; Affective states were assessed with one item per affective state: fear, happiness, and anger (7-point Likert)	The highest level of PTG was found in participants who reported negative–moderate feelings (moderate levels of anger, fear, and happiness) during the first lockdown.	not reported
Hyun et al. (2021) [44]	longitudinal study	US, young adults (N = 805)	T1: April to August 2020 T2: September 2020 to March 2021	24.8	84.8% females 11.3% males 3.9% other	PTGI-SF COVID-19-adapted	Connor–Davidson Resilience Scale (CD-RISC-10); Distress Tolerance Scale (DTS); Family Connectedness Scale (FCS); Patient Health Questionnaire (PHQ-8); Generalized Anxiety Disorder Scale (GAD-7); PTSD Checklist—Civilian Version (PLC-C); developed 6-item measure for pandemic-related worry (5-point Likert)	PTSD symptoms and COVID-19-related worries significantly predicted higher levels of PTG, while depression symptoms predicted lower levels of PTG; resilience and family connectedness significantly predicted higher levels of PTG; distress tolerance significantly predicted lower levels of PTG; Asians were less likely to report PTG.	not reported
Hyun et al. (2023) [45]	longitudinal study	US, young adults (N = 661)	T1: April to August 2020 T2: September 2020 to March 2021 T3: April to May 2021	25.4	85.3% females 14.7% males	PTGI-SF COVID-19-adapted	Pandemic-related distress measure consisted of 14 items capturing four areas of distress: financial stress, COVID-19 health risk, COVID-19-related worries, COVID-19-related grief; Patient Health Questionnaire (PHQ-8); seven-item Generalized Anxiety Disorder Scale (GAD-7)	At low levels of pandemic-related distress, depressive symptoms were similar for young adults with low, moderate, or high PTG; at high levels of pandemic-related distress, young adults with low PTG had the highest levels of depressive symptoms, and young adults with high PTG had the lowest levels of depressive symptoms; PTG at T2 buffered the effect of COVID-19-related distress from 2020 on depressive symptoms in 2021 among US young adults.	not reported
Ikizer et al. (2021) [46]	cross-sectional study	Turkey, general population (N = 685)	between 17 June and 21 August 2020 (new cases in Turkey had plateaued between June and August at around 1000 a day)	34.6	63.6% females 34.6% males 1.8% other	PTGI 21-item COVID-19-adapted	Six questions were administered to assess the severity of COVID-19 exposure; The Event-Related Rumination Inventory (ERRI); 10-item Perceived Stress Scale (PSS); PTSD Checklist for Diagnostic and Statistical Manual of Mental Disorders, Fifth Edition (PCL-5); Post-Traumatic Growth Inventory-42 (21 items for Post-Traumatic Depreciation)	Positive correlation between PTS and PTG; positive correlation between PTG and PTD higher PTG was associated with lower levels of education and anticipating financial risks as a result of the pandemic; engaging in deliberate rumination emerged as another predictor of PTG.	not reported
Kalaitzaki and Tamiolaki (2022) [47]	cross-sectional study	Greece, general population (N = 1361)	5–30 April 2020 (during the first COVID-19 lockdown) and 15 November to 12 December 2020 (during the second lockdown)	35.7	77.6% females 22.4% males	PTGI 21-item COVID-19-adapted	Post-Traumatic Stress Disorder Checklist for DSM-5 (PCL-5); Brief Coping Orientation to Problems Experienced Inventory (COPE); The ENRICHD Social Support Instrument (ESSI)	PTG did not significantly increase during the second lockdown; PTG was associated with PTSS during the first lockdown and with perceived stress during the second one; both adaptive and maladaptive coping strategies predicted PTG during the first lockdown, whereas only adaptive coping strategies predicted PTG during the second lockdown; perceived social support, emotional during the first lockdown, and instrumental during the second one, predicted PTG during the two lockdowns, respectively.	more than half of the participants displayed PTG during both lockdowns, with a trend of higher rates in the second lockdown (from 52.7% to 55.1%)
Landi et al. (2022) [48]	longitudinal study	Italy, general population (N = 382)	T1: 9–19 July 2020 (three months after the first national Italian lockdown, a period of better control of the pandemic in which all restrictions were lifted) T2: 9–19 October 2020 (the number of new COVID-19 cases started to increase again but with no corresponding restrictions)	40.5	77.5% females 22.5% males	PTGI-SF COVID-19-adapted	30-item Multidimensional Psychological Flexibility Inventory (MPFI); Impact of Event Scale-Revised (IES-R)	Time 1 PTG exhibited a significant small correlation with Time 1 PTS and a strong correlation with Time 2 PTG; Time 2 PTS was significantly and positively correlated with Time 2 PTG; being older was negatively correlated with Time 2 PTG while being infected with COVID-19 over the study period was positively associated with Time 2 PTG; higher PTG scores emerged in the high-PTS group; higher psychological flexibility at Time 1 and four of its subprocesses (present moment awareness, defusion, values, and committed action) were associated with higher PTG at Time 2 among people in the high-PTS group (but not in the low PTS group).	most (69.01%) participants did not report moderate-to-high PTG in any domain in the low PTG group, while in the high PTG group, 53.14% and 28.14% reported growth on at least one or two PTGI-SF domains, respectively.
Laslo-Roth et al. (2022) [49]	cross-sectional study	Israel, general population (N = 275)	from 15 March to 15 April 2020 (participants were subjected to social-distancing regulations during this period)	33.4	78.2% females 21.8% males	PTGI 21-item COVID-19-adapted	Questionnaire by the National Organization on Disability (NOD) with five items representing social participation: interactions with friends and family, religious activity, participating in social events in the community, volunteer activities or public activities, and going out to parks in the community; Adult state hope scale 6-items; Multidimensional scale of perceived social support 12 items; Cognitive reappraisal subscale of the Emotion Regulation Questionnaire (ERQ)	Agency thinking predicted PTG only indirectly, through social support and cognitive reappraisal; perceived social support was identified as a mediating factor between social participation and PTG; the perception of oneself in ordinary times as being socially engaged played a major role in the perception of psychological growth during the pandemic, and social participation was found to be linked directly and indirectly, through hope, to PTG.	not reported
Lau et al. (2021) [50]	longitudinal study	China, general population (N = 327)	T1: from 12 March to 8 April 2020 (first major wave of a local outbreak in Hong Kong; mandatory closure of premises and businesses, and catering restrictions in restaurants: reduced service capacity and mandatory intertable distance in late March) T2: from 24 April to 12 May 2020 (palliation of the outbreak, only one case per day)	35	71.9% females 28.1% males	PTGI 21-item COVID-19-adapted	The perceived severity of the COVID-19 outbreak was measured by two items (10-point Likert); 13-item SOC (SOC-13); 22-item Impact of Event Scale-Revised (IES-R)	Having an above-sample-median monthly household income (HKD 40,000), being tertiary educated, and being with a family member or close friend who has experienced medical quarantine or having tested positive for COVID-19 were related to a higher likelihood of PTG; PTG was more likely to emerge in participants with high levels of both SOC and PTS: the interaction between the SOC and PTS mediated the relationship between Time 1 perceived outbreak severity and Time 2 PTG, such that PTG was more likely among participants with higher PTS and SOC; PTG was also associated with a weaker contingency between Time 1 and Time 2 perceived outbreak severity.	1.8% attained substantial PTG; 18.0% reported significant PTG in at least one domain in Time 2
Lewis et al. (2022) [51]	longitudinal study	England and Wales, adults with lived experience of a psychiatric disorder (N = 1424)	T1: June to July 2020 T2: November 2020 to January 2021	46.7	75.3% females 21.9% males 2.3% other	PTGI-SF COVID-19-adapted	COVID-19-related information and social support were measured by asking participants whether they or anyone close to them had experienced symptoms of COVID-19 and whether they had tested positive (“yes” or “no”); Participants were asked how socially supported they felt by friends and family in the past 2 weeks (5-point Likert); COVID-19-related trauma exposure was measured by asking participants if they found any aspect of the COVID-19 crisis traumatic (“yes” or “no”). If they answered “yes,” they were prompted to give a free-text description of their most troubling COVID-19-related experience; International Trauma Questionnaire (ITQ); WHO-5 Well-Being Index (WHO-5)	Higher PTGI-SF scores were most strongly associated with increased perceived social support, and perception of the pandemic as being traumatic; in the subsample of participants who perceived the pandemic as traumatic and completed the ITQ, higher PTGI-SF scores were most strongly associated with higher levels of psychological well-being and more severe PTSD symptoms.	not reported
Li and Hu (2022) [52]	cross-sectional study	China, college students from universities (N = 463)	between 18 May and 22 July 2021	not reported	78.8% females 21.2% males	PTGI 21-item adapted	Connor–Davidson Resilience Scale (CD-RISC); Simplified Coping Style Questionnaire (SCSQ); Emotion Regulation Questionnaire; Awareness and impact of COVID-19 were collected through three questions (5-point Likert)	Psychological Resilience (PR), Positive Coping (PC), Cognitive Reappraisal (CR), and PTG were positively correlated with each other; PTG had a direct and positive association with PR, PC, and CR, respectively, mediated the relationship between PTG and PR; students with high-level PTG tended to report increased use of PC, which further facilitated their CR and, subsequently, promoted their PR.	not reported
Matos et al. (2021) [53]	cross-sectional study	21 countries worldwide (Europe, North America, South America, Asia, Oceania, Middle Est), general population (N = 4057)	between mid-April and mid-May 2020 (early months of the COVID-19 pandemic)	41.5	80.8% females 18.2% males 0.4% other 0.6% preferred not to report their gender	PTGI 21-item COVID-19-adapted	Social Connection: Compassionate Engagement and Action Scales (CEAS) and Social Safeness and Pleasure Scale (SSPS); Social Disconnection: Fears of Compassion Scales (FCS) and UCLA Loneliness Scale (UCLA LS); Perceived Coronavirus Risk Scale (PCRS); Impact of Event Scale-Revised (IES-R)	Higher perceived threat of COVID-19 predicted greater PTG; social connection (compassion and social safeness) received from others was a significant predictor of PTG; the interaction effect of perceived threat of COVID-19 and the social connection component was significant and positive, indicating that the three flows of compassion and social safeness significantly moderate (magnify) the impact of fear of contraction on PTG; that fears of compassion and loneliness significantly moderate (reduce) the impact of fear of contraction on PTG.	not reported
Na et al. (2021) [54]	prospective cohort study (with cross-sectional data)	US, military veterans (N = 3078)	Wave 1: median completion date on 21 November 2019 (pre-pandemic survey, before the first documented COVID-19 case in the U.S) Wave 2: median completion date: 14 November 2020 (peri-pandemic survey)	63.3	8.4% females 91.6% males	PTG-SF COVID-19-adapted	Responses on the two depressive symptoms of the PHQ-4; responses on the two generalized anxiety items of the PHQ-4; Life Events Checklist for DSM-5; Adverse Childhood Experiences Questionnaire; sum of the number of medical conditions endorsed in response to two questions; 10-Item Personality Inventory; endorsement of current treatment with psychotropic medication and/or psychotherapy or counseling in response to two questions; Duke University Religion Index; Medical Outcomes Study Social Support Scale-5; Connor-Davidson Resilience Scale-10; Number of close friends and family members; Secure attachment: response to one question; Purpose in Life Test-Short Form; a single-item measure of optimism from Life Orientation Test-Revised; single-item measure of gratitude from Gratitude Questionnaire; 5-item version of the Medical Outcomes Study Social Support Scale; single-item from Curiosity and Exploration Inventory-II; Perceived level of community integration (one question, 7-point Likert); Change variables from pre-pandemic to peri-pandemic; 4-item PTSD Checklist for DSM-5; total count of past-year potentially traumatic events	Female gender, non-white ethnicity, agreeableness, and protective psychosocial characteristics (purpose in life, religiosity/spirituality, and PTG in relation to earlier trauma) were related to PTG; pandemic-related factors associated with PTG included pandemic-related worries (physical health, mental/emotional health), social restriction stress, stress related to changes in family contacts, stress related to changes in social contacts, financial difficulties, stability of living situation, and PTSD symptoms; worries about the effect of the pandemic on one’s physical and mental health, PTG in response to pre-pandemic traumatic life events, and greater severity of pandemic-related avoidance symptoms were the strongest correlates of pandemic-related PTG.	not reported
Northfield and Johnston (2022) [55]	cross-sectional study	US, general population (N = 296)	August 2020	39.7	58.8% females 41.2% males	PTGI 21-item COVID-19-adapted	The Impact of Event Scale-Revised (IES-R); Multidimensional Scale of Perceived Social Support	A strong positive relationship was found between psychological distress and growth; this relationship was moderated by social support such that the relationship was stronger at higher levels of social support; perceived social support from significant others was not a strong predictor of PTG; however, perceived support from family and friends were; age significantly predicted PTG, with those younger in age reporting higher levels of PTG.	33.4% of the sample scored 63 or higher which is indicative of a moderate growth
Pietrzak et al. (2022) [56]	prospective cohort study (with cross-sectional data)	US, military veterans (N = 3078)	Wave 1: between 18 November 2019 and 8 March 2020 Wave 2 (1-year follow-up): between 9 November and 19 December 2020	63.3	8.4% females 91.6% males	PTGI-SF COVID-19-adapted	Survey with pandemic-associated risk factors (also PTSD symptoms), background characteristics (also suicidal ideation)	Veterans who screened positive for COVID-19-associated PTSD symptoms had a markedly higher prevalence of PTG; greater COVID-19-associated improvements in appreciation of life and social relationships were associated with a significant reduction in the odds of suicidal ideation.	43.3% reported moderate or greater levels of PTG
Sandrin et al. (2022) [57]	cross-sectional study	France, working population (N = 2004)	October 2020 (prior to the second confinement in France)	range: 18–60+	48% females 52% males	PTGI 21-item COVID-19-adapted	Psychosocial safety climate was measured with four items (5-point Likert); Performance was measured by responses to the following question: “Over the past week, how would you rate your performance at work on a scale of 0–100%? ”; Kessler Psychological Distress Scale (K6)	Mediation analyses indicate that the psychosocial safety climate at work place has a direct and positive influence on PTG and performance, as well as a direct negative influence on psychological distress.	not reported
Shigemoto (2022) [58]	longitudinal study	US, Amazon’s Mechanical Turk (N = 71)	the daily survey started on 3 May and ended on 31 May 2020 (at that time, in the U.S, there have been 1,150,000 cases of COVID-19 and 67,000 deaths, and each day, 30,000 new cases of COVID-19 have been reported)	41.9	46.5% females 53.5% males	PTGI-SF COVID-19-adapted	Event-Related Rumination Inventory (ERRI)	No significant relation was found between intrusive rumination and PTG, but there was a statistically significant positive association between deliberate rumination and PTG.	not reported
Wall et al. (2023) [59]	cross-sectional study	UK, general population (N = 440)	May and June 2020 (during lockdown)	not reported	91.8% females 7.7% males 0.5% unknown	PTGI 21-item COVID-19-adapted	Impact of event scale-revised (IES-R); Brief COPE scale; Multidimensional Scale of Perceived Social Support (MSPSS); Connor-Davidson Resilience Scale-10 (CD-RISC-10); Life Orientation Test-Revised (LOT-R); Event-Related Rumination Inventory (ERRI)	Centrality of event, deliberate rumination, and social support were significant predictors of PTG.	49.5% of the sample scores of 45 or above on the PTGI (high levels of PTG)
Wang and Huang (2022) [60]	cross-sectional study	China, college students from a university (N = 690)	not reported	not reported	64.5% males 35.5% females	PTGI-13 item COVID-19-adapted	Self-efficacy scale comprising four dimensions and 15 items: tolerance ambiguity self-efficacy, opportunity-identification self-efficacy, relationship self-efficacy, and managerial self-efficacy (5-point Likert); Prosocial tendency Scale revised; Entrepreneurial intention scale comprising two dimensions (namely, goal intentions and implementation intentions) and 10 items (7-point Likert)	PTG significantly and positively affects the entrepreneurial intention of Chinese college students who have experienced trauma due to the COVID-19 pandemic; entrepreneurial self-efficacy and prosocial tendencies have a chain mediating effect on the relation between PTG and entrepreneurial intention.	not reported
Xiao et al. (2022) [61]	cross-sectional study	China, patients recovered from COVID-19 who were discharged from hospitals (N = 199)	from August to September 2020, discharged patients with COVID-19 are centralized and quarantined for 14 days in designated facilities and then quarantined for another 14 days at home	42.7	53.3% females 46.7% males	PTGI COVID-19-adapted	Questionnaire developed for this study: Demographic and Pre-hospitalization variables, Hospitalization variables, Post-hospitalization variables, Perceived Discrimination (nine questions, “yes”/”no”), Perceived Affiliate Stigma (seven questions, 4-point Likert), Perceived Impact of being Infected with COVID-19 (three questions, 10-point Likert), Social support (four questions, 10-point Likert); Patient Health Questionnaire (PHQ-15); Self-Stigma Scale; 2-item Connor–Davidson Resilience Scale (CD-RISC2); Patient Health Questionnaire (PHQ-9); 7-item Generalized Anxiety Disorder (GAD-7)	Having children, receiving mental healthcare services during hospitalization, clinical classification of COVID-19 at entry, self-stigma, and social support were significantly associated with PTG.	not reported
Xie and Kim (2022) [62]	cross-sectional study	Mainly Sweden and China (other countries 16.6%), general population (N = 181)	from 25 April to 5 May 2021	24.7	70.2% females 29.8% males	PTGI-S COVID-19-adapted	Multidimensional Scale of Perceived Social Support (MSPSS); Ten-Item Personality Inventory (TIPI); Brief COPE Questionnaire	Perceived social support, personality traits (extraversion, emotional stability, agreeableness, and conscientiousness), and coping strategies (problem-focused coping, emotion-focused coping, and social support coping) were positively correlated with PTG; coping strategies (problem-focused coping, emotion-focused coping, and avoidance coping) mediated the relations between perceived social support, personality traits, and PTG	60.8% of participants with scores of 32 points or higher demonstrated personal growth
Xie et al. (2022) [63]	longitudinal study	China, self-quarantined college students (N = 226)	T1: 27 February 2020, with a 1-week duration (the peak phase of the COVID-19 in China, all universities in China have suspended offline classes and students have been asked to stay confined at home T2: 5 May 2021, with a 2-week duration (the post-pandemic phase in China)	19.6	41.2% females 58.8% males	PTGI 21-item COVID-19-adapted	Satisfaction with Life Scale (SWLS); Personal Values Questionnaire (PVQ)	PTG at the peak phase of the COVID-19 pandemic was positively associated with subsequent LS (Life Satisfaction). One year later, the association between COVID-19-related PTG at Time 1 and LS at Time 2 was partially mediated by ST (self-transcendence) and SE (self-enhancement) values at Time 2: PTG at Time 1 was positively related to ST value while negatively related to SE value at Time 2.	moderate to high levels of PTG; 65.4% of participants experienced at least medium levels of positive changes
Yan et al. (2021) [64]	cross-sectional study	China, discharged COVID-19 patients (N = 140)	February 2020	43.5	53.6% females 46.4% males	PTGI 21-item adapted	Profile of Mood Status (POMS); Post-Traumatic Stress Disorder Self-Rating Scale (PTSD-SS); Simplified Coping Style Questionnaire (SCSQ); Multidimensional Scale of Perceived Social Support (MSPSS)	Lower levels of mood disturbance, more severe PTSD, more positive coping style, and more perceived social support were associated with a higher level of PTG; PTG was negatively related to anger and time from onset to diagnosis in discharged COVID-19 patients; self-esteem showed a significant correlation with PTG.	not reported

Legend. CR: cognitive reappraisal; ITQ: International Trauma Questionnaire; LS: life satisfaction; PC: positive coping; PR: psychological resilience; PTD: post-traumatic depreciation; PTG: post-traumatic growth; PTGI-SF: Post-Traumatic Growth Inventory—Short Form; PTS: post-traumatic stress; PTSD: post-traumatic stress disorder; PTSS: post-traumatic stress symptoms; SE: self-enhancement.

**Table 2:** Risk of bias (ROB) assessment of the included studies.

Author	Were the Criteria for Inclusion in the Sample Clearly Defined?	Were the Study Subjects and the Setting Described in Detail?	Was the Exposure Measured in a Valid and Reliable Way?	Were Objective Standard Criteria Used for Measurement of the Condition?	Were Confounding Factors Identified?	Were Strategies to Deal with Confounding Factors Stated?	Were the Outcomes Measured in a Valid and Reliable Way?	Was Appropriate Statistical Analysis Used?	Number of YES	ROB
Bayless (2021) [36]	Yes	Yes	Yes	Not Applicable	Yes	Yes	Yes	Yes	7	Low
Casali et al. (2021) [37]	No	Yes	Yes	Not Applicable	Yes	Yes	Yes	Yes	6	Low
Celdrán et al. (2021) [38]	No	Yes	Yes	Not Applicable	No	No	No	Yes	3	Moderate
Chasson et al. (2022) [39]	Yes	Yes	Yes	Not Applicable	No	No	No	Yes	4	Moderate
Chen and Tang (2021) [40]	Yes	Yes	Yes	Not Applicable	No	No	Yes	Yes	5	Moderate
Ellena et al. (2021) [41]	No	Yes	Yes	Not Applicable	No	No	No	Yes	3	Moderate
Fino et al. (2022) [42]	Yes	Yes	Yes	Not Applicable	Yes	Yes	Yes	Yes	7	Low
Goutaudier et al. (2022) [43]	Yes	Yes	Yes	Not Applicable	No	No	Yes	Yes	5	Moderate
Hyun et al. (2021) [44]	No	No	Yes	Not Applicable	No	No	Yes	Yes	3	Moderate
Hyun et al. (2023) [45]	Yes	Yes	Yes	Not Applicable	No	No	Yes	Yes	5	Moderate
Ikizer et al. (2021) [46]	No	Yes	Yes	Not Applicable	No	No	Yes	Yes	4	Moderate
Kalaitzaki and Tamiolaki (2022) [47]	No	Yes	Yes	Not Applicable	No	No	Yes	Yes	4	Moderate
Landi et al. (2022) [48]	Yes	Yes	Yes	Not Applicable	No	No	Yes	Yes	5	Moderate
Laslo-Roth et al. (2020) [49]	No	Yes	Yes	Not Applicable	No	No	Yes	Yes	4	Moderate
Lau et al. (2021) [50]	Yes	Yes	Yes	Not Applicable	No	No	Yes	Yes	5	Moderate
Lewis et al. (2022) [51]	Yes	Yes	Yes	Not Applicable	No	No	Yes	Yes	5	Moderate
Li and Hu (2022) [52]	Yes	Yes	Yes	Not Applicable	Unclear	Unclear	Yes	Yes	5	Moderate
Matos et al. (2021) [53]	No	Yes	Yes	Not Applicable	No	No	Yes	Yes	4	Moderate
Na et al. (2021) [54]	No	No	Yes	Not Applicable	No	No	Yes	Yes	3	Moderate
Northfield and Johnston (2021) [55]	Yes	Yes	Yes	Not Applicable	No	No	Yes	Yes	5	Moderate
Pietrzak et al. (2022) [56]	No	No	Yes	Not Applicable	Unclear	Unclear	Yes	Yes	3	Moderate
Sandrin et al. (2022) [57]	Yes	Yes	Yes	Not Applicable	No	No	Yes	Yes	5	Moderate
Shigemoto (2022) [58]	Yes	Yes	Yes	Not Applicable	No	No	Yes	Yes	5	Moderate
Wall et al. (2023) [59]	No	Yes	Yes	Not Applicable	No	No	Yes	Yes	4	Moderate
Wang and Huang (2022) [60]	Yes	Yes	Yes	Not Applicable	No	No	Yes	Yes	5	Moderate
Xiao et al. (2022) [61]	Yes	Yes	Yes	Not Applicable	Unclear	Unclear	Yes	Yes	5	Moderate
Xie and Kim (2022) [62]	Yes	Yes	Yes	Not Applicable	No	No	Yes	Yes	5	Moderate
Xie et al. (2022) [63]	No	Yes	Yes	Not Applicable	No	No	Yes	Yes	4	Moderate
Yan et al. (2021) [64]	Yes	Yes	Yes	Not Applicable	No	No	Yes	Yes	5	Moderate

## Data Availability

As a systematic review, this study did not involve any original data.

## Supplementary Materials

The following supporting information can be downloaded at: https://www.mdpi.com/article/10.3390/jcm13010095/s1, S1: Search strategies; S2: PRISMA flow diagram.
