# Supplementary material for: Factors Associated with Post-Traumatic Growth during the COVID-19 Pandemic: A Systematic Review
Source: J Clin Med. 2023 Dec 23;13(1):95. doi: 10.3390/jcm13010095 (PMC10779553; doi:10.3390/jcm13010095)

S1. Search strategies

PUBMED

("Posttraumatic Growth, Psychological"[Mesh] OR posttraumatic-growth*[tiab] OR PTG[tiab] OR post-traumatic-growth*[tiab] OR stress-related-growth*[tiab]) AND ("COVID-19"[Mesh] OR "SARS-CoV-2"[Mesh] OR ("COVID-19"[Supplementary Concept] OR "severe acute respiratory syndrome coronavirus 2"[Supplementary Concept] OR SARS-COV-2[tiab] OR severe-acute-respiratory-syndrome-coronavirus-2[tiab] OR SARS-Coronavirus-2[tiab] OR sars-cov-2[tiab] OR SARS2[tiab] OR nCoV[tiab] OR novel-coronavirus[tiab] OR novel-corona-virus[tiab] OR new-coronavirus[tiab] OR new-corona-virus[tiab] OR 2019nCoV[tiab] OR 2019-nCoV[tiab] OR covid-19[tiab] OR coronavirus-disease-2019[tiab] OR corona-virus-disease-2019[tiab] OR coronavirus-disease-19[tiab] OR corona-virus-disease-19[tiab] OR COVID*[tiab] OR HCoV-19[tiab]))

EMBASE

('posttraumatic growth (psychology)'/exp OR 'posttraumatic growth inventory'/exp OR 'posttraumatic growth*':ti,ab OR 'post traumatic growth*':ti,ab OR 'positive change*':ti,ab OR ptg*:ab,ti) AND ('coronavirus disease 2019'/exp OR 'severe acute respiratory syndrome coronavirus 2':ti,ab,kw OR 'sars coronavirus 2':ti,ab,kw OR 'sars cov 2':ti,ab,kw OR sars2:ti,ab,kw OR ncov:ti,ab,kw OR 'novel coronavirus':ti,ab,kw OR 'novel corona virus':ti,ab,kw OR 'new coronavirus':ti,ab,kw OR 'new corona virus':ti,ab,kw OR 2019ncov:ti,ab,kw OR '2019 ncov':ti,ab,kw OR 'covid 19':ti,ab,kw OR 'coronavirus disease 2019':ti,ab,kw OR 'corona virus disease 2019':ti,ab,kw OR 'coronavirus disease 19':ti,ab,kw OR 'corona virus disease 19':ti,ab,kw OR covid*:ti,ab,kw OR 'hcov 19':ti,ab,kw OR 'sars-related coronavirus'/exp OR 'severe acuterespiratory syndrome' OR 'acute respiratory syndrome*':ti,ab,kw OR sars:ti,ab,kw OR 'sars related':ti,ab,kw OR 'sars cov':ti,ab,kw OR 'sars associated':ti,ab,kw OR 'sars co v':ti,ab,kw OR 'middle east respiratory syndrome coronavirus'/exp OR 'middle east respiratory syndrome'/exp OR 'middle east respiratory syndrome*':ti,ab,kw OR mers:ti,ab,kw OR 'mers cov':ti,ab,kw OR 'mers co v':ti,ab,kw)

PSYCINFO

(DE "Posttraumatic Growth" OR TI posttraumatic growth* OR AB post-traumatic-growth* OR TI positive change* OR AB positive change* OR TI PTG OR AB PTG OR TI stress-related growth* OR AB stress-related growth*) AND ((TI SARS-COV-2 OR TI severe-acute-respiratory-syndrome-coronavirus-2 OR TI SARS-Coronavirus-2 OR TI sars-cov-2 OR TI SARS2 OR TI nCoV OR novel-coronavirus OR TI novel-corona-virus OR TI new-coronavirus OR TI new-corona-virus OR TI 2019nCoV OR TI 2019-nCoV OR TI covid-19 OR TI coronavirus-disease-2019 OR TI corona-virus-disease-2019 OR TI coronavirus-disease-19 OR TI corona-virus-disease-19 OR TI COVID* OR TI HCoV-19 OR TI acute-respiratory-syndrome* OR TI SARS OR TI SARS-related OR TI SARS-CoV OR TI SARS-associated OR TI SARS-Co-V OR DE "COVID-19" OR DE "Middle East Respiratory Syndrome" OR AB SARS-COV-2 OR AB severe-acute-respiratory-syndrome-coronavirus-2 OR AB SARS-Coronavirus-2 OR AB sars-cov-2 OR AB SARS2 OR AB nCoV OR AB novel-coronavirus OR AB novel-corona-virus OR AB new-coronavirus OR AB new-corona-virus OR AB 2019nCoV OR AB 2019-nCoV OR AB covid-19 OR AB coronavirus-disease-2019 OR AB corona-virus-disease-2019 OR AB coronavirus-disease-19 OR AB corona-virus-disease-19 OR AB COVID* OR AB HCoV-19 OR AB acute-respiratory-syndrome* OR AB SARS OR AB SARS-related OR AB SARS-CoV OR AB SARS-associated OR AB SARS-Co-V))

S2. PRISMA Flow Diagram

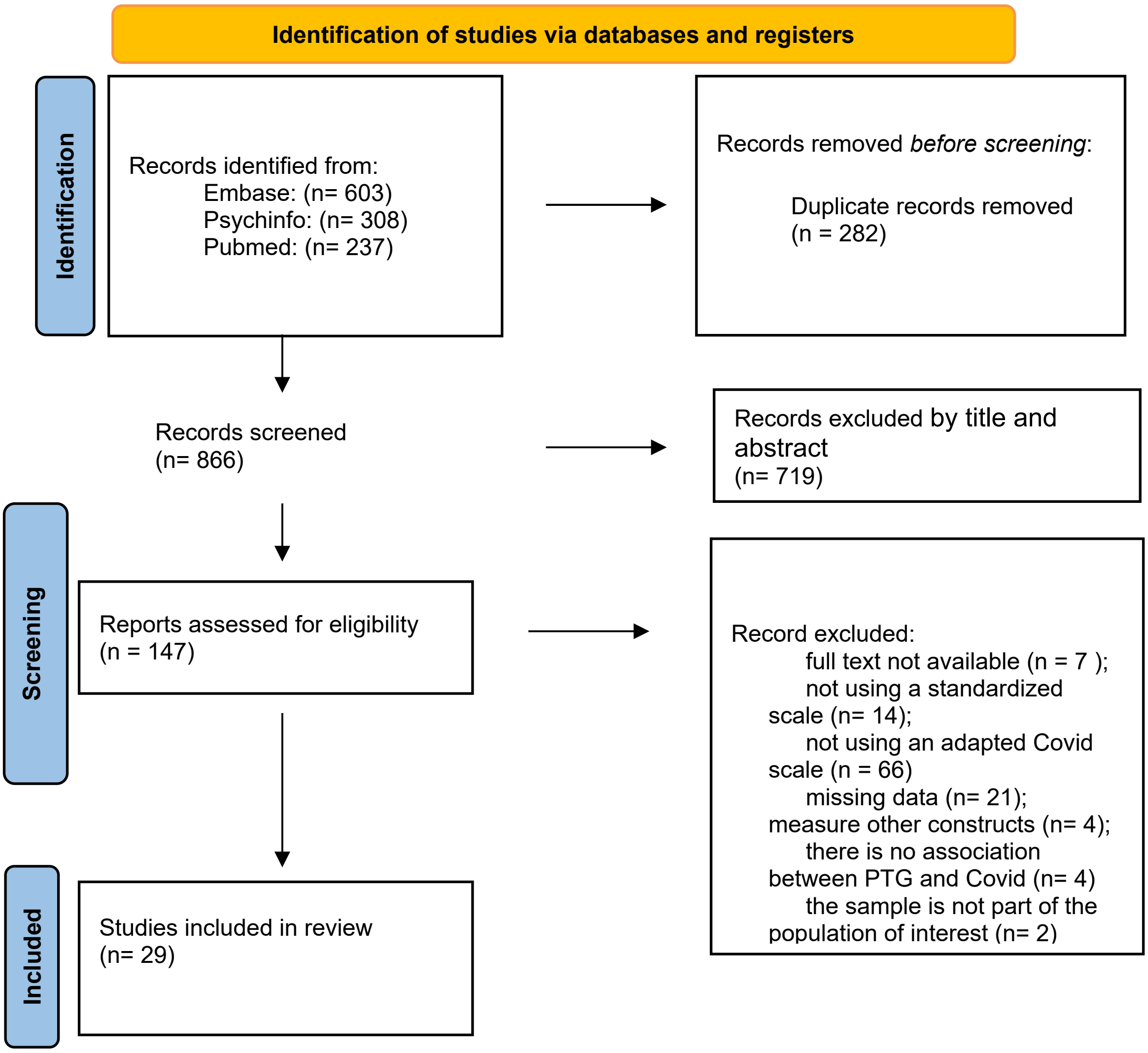

Supplement: Supplementary file 1 [file jcm-13-00095-s001.zip › jcm-2758059-supplementary.pdf]
